# Political Pressures Increased Vulnerability to Climate Hazards for Nomadic Livestock in Inner Mongolia, China

**DOI:** 10.1038/s41598-017-08686-4

**Published:** 2017-08-15

**Authors:** Ang Li, Shi Chen, Xueyao Zhang, Jianhui Huang

**Affiliations:** 1 State Key Laboratory of Earth Surface Processes and Resource Ecology, Beijing Normal University, Beijing, 100875 China; 2 State Key Laboratory of Vegetation and Environmental Change, Institute of Botany, the Chinese Academy of Sciences, Beijing, 100093 China; 3 Department of Public Health Sciences, University of North Carolina Charlotte, Charlotte, USA 28223; 4 Data Science Initiative, University of North Carolina Charlotte, Charlotte, 28223 USA; 5 University of Chinese Academy of Sciences, Beijing, 100094 China

## Abstract

Herders in Inner Mongolia experienced two completely different political periods during their last nomadic period between 1961 and 1986. However, climate and technical factors were very similar between these two periods according to statistical analyses. We retrieved historical climate and livestock population data and performed a retrospective study using generalized additive models to analyze three major livestock population demographic metrics changes between these two periods. We found that the sociopolitical factors significantly impacted all three major demographic metrics (adult mortality, neonatal mortality and birthrate) between the two periods for both large (cattle, horse, and camel) and small livestock (sheep and goat). We also identified the interaction effects between sociopolitical factor and climate factors for adult and neonatal mortality, while birthrate was not affected by these interactions. When exposed to climate hazards, adult and neonatal livestock mortality rates were significantly higher, while birthrate was significantly lower in social movement period than in peaceful period. We concluded that political movements had indeed increased the vulnerability of herders’ livestock to climate hazards. External political pressures deprived hazard-resistance entitlements of herders, which may explain the elevated effects of political pressures on livestock vulnerability.

## Introduction

Many human societies are vulnerable to climate extremes and are also under pressure from socialeconomic and political forces^1^. Herders are especially vulnerable to inclement weather and climate extremes, as they generally live in marginal lands around the world^2^, such as grasslands and mountain areas, where climate is usually more unpredictable^3^. Warm and wet climate usually facilitates a plentiful livestock harvest^4, 5^, whereas blizzards and droughts not only have threatened herders’ livestock (e.g., increasing mortality) but also herders’ own lives^6^. Nomadic livestock populations frequently crash during anomalously cold winters in pasture regions of Inner Asia, such as Mongolia, Kazakhstan, and Inner Mongolia in China^7–10^. Based on the correlations between long-term climate data and nomadic historical events^11^, scholars have argued that climate change is able to explain the history of nomadic herders adequately well. Many studies with qualitative methods have demonstrated correlations between optimal climate and the rising of nomadic nations^4, 11^. On the other hand, these qualitative studies have also found that climate hazards often trigger production failures, wars, and even collapse of nomadic nations^5, 12^.

Climate hazard is just one component of the vulnerability for the socio-ecological system, where people can anticipate, cope with, and resist the adverse impacts of climate^13, 14^. However, external political pressures may impede the ability of people to cope with climate hazards and adapt to climate changes^15^. Entitlement approach, which plays an important role in vulnerability theory framework, explains why vulnerability of human groups to climate hazards significantly increases under political pressures^14, 15^. The original entitlement approach was developed from famine studies. It revealed that famine was not solely due to agriculture failure caused by climate disasters, but rather from loss of food obtaining entitlements, which included freedom in production, harvest, and trade^16^. Similarly, herders are usually limited in maintaining the mobility of livestock and flexibility of grassland use by the government’s political decisions, such as privatization and fencing of grasslands^17, 18^. Consequently, herders lose their entitlements to raise their animals in proper and traditional ways, which would increase their vulnerability to climate disasters^17–20^.

There are some methodological advantages and shortcomings in both abovementioned qualitative and quantitative studies. Quantitative methods can analyze historical data on long-term temporal and broad spatial scales. However, these methods seldom address influences from the socialpolitical perspective, which is difficult to quantify. Thus, these studies usually reveal correlations rather than causations. On the other hand, qualitative methods, such as context analysis, field surveys, and interviews, can consider how climatic and multiple socialpolitical factors interact. However, field surveys and interviews are usually limited to a small number of sites as well as small sample sizes. Furthermore, herders’ memory and thinking about climatic and social events may change among generations^21^, while the context about nomadic herders is usually incomplete.

More robust quantitative methods have been developed in econometrics and cliometrics. The general steps of these methods start from a group of competing hypotheses, then using statistical methods for data analysis, and evaluating whether to accept a hypothesis based on certain statistics. This quantitative method can further integrate social-political information as well as long-term and broad historical data.

Herders in Inner Mongolia are a great example to study the complicated interactions between sociopolitical and climate factors^22, 23^, and their impacts on vulnerability of nomadic livestock. During the period of socialism planned economy (1947–1986), the Chinese government established a statewide, comprehensive, and modern census system in 1961^9^. Although the government had gradually introduced modern machinery and a communal economic system into urban areas of Inner Mongolia, herders had maintained their nomadic traditions and fought against adverse climates, such as blizzards and droughts, with their traditional knowledge and less developed infrastructure. This period was the “last nomadic period” of Inner Mongolia^3, 24^.

There were two distinct sociopolitical periods during this last nomadic period, differing mostly in political pressures. During 1964–1978, social movements (the Great Cultural Revolution) continued to disturb the livestock husbandry. Under heavy political pressures, officials established many policies regardless of the effects of these policies, such as “grassland reclamation” and “grain self-sufficiency” policies^25^. Local herders lost thee freedom of actively raise, sell, breed, and move their livestock^8^. The other period (before 1964 as well as between 1979 and 1986) was much more peaceful (thus called peaceful period hereafter). Herders, along with grassroots officials and local elites, had adequate freedom in animal husbandry. Herders also coped with climate hazards following the guidelines of their traditional knowledge in the peaceful period, which co-evolved thousands of years along with their environment^9, 17, 26^.

**Figure 1:**
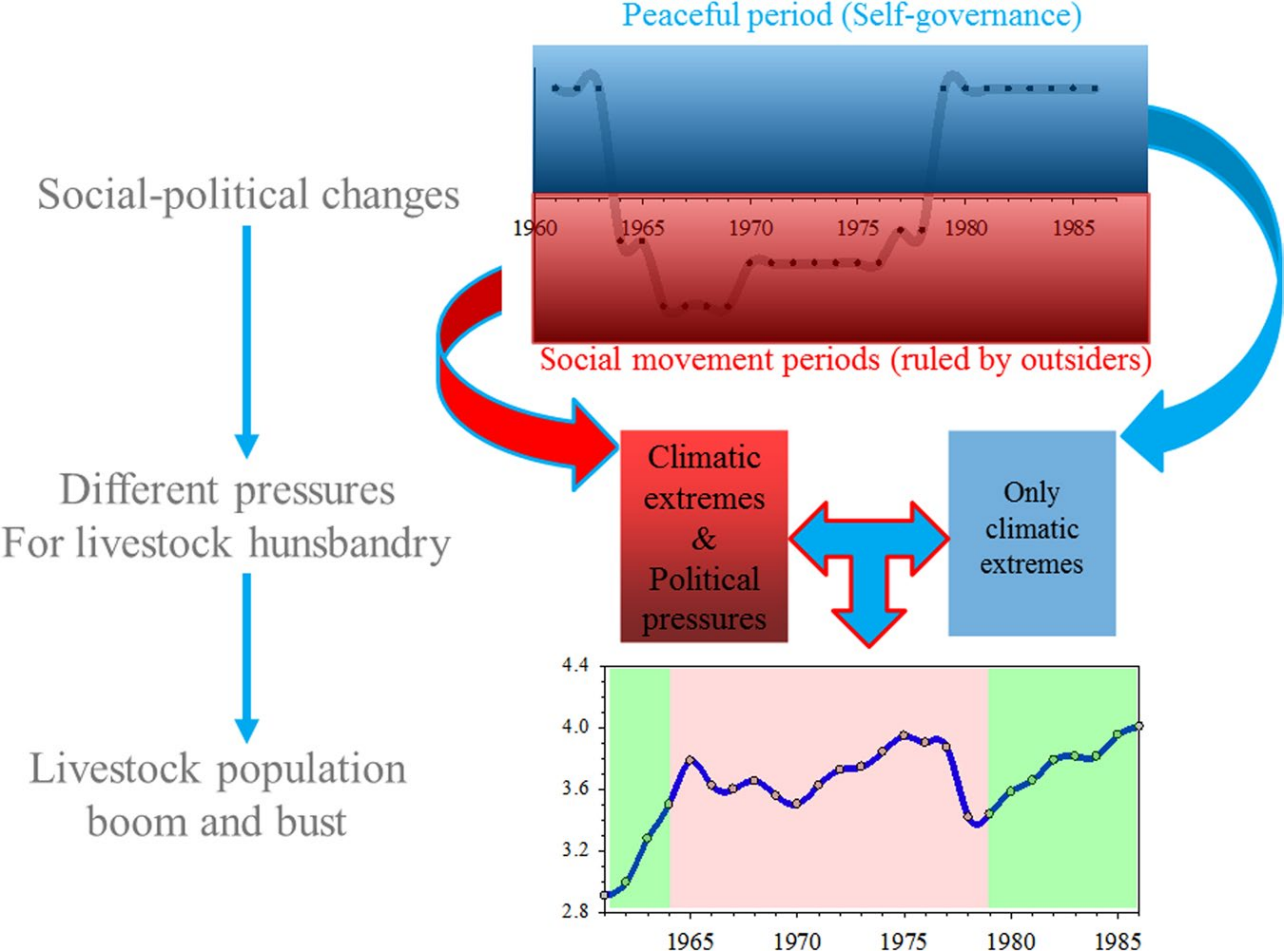
The conceptual diagram of sociopolitical pressure change impacted the local animal husbandry managements and livestock population dynamics. Note: The political regime can be divided into peaceful (1961–1963, 1979–1986) and social movements period (1964–1978) based on sociopolitical pressures.

Besides the difference in sociopolitical pressures, other climate and social factors were less distinct between these two periods, including the frequency of climate extremes, livestock species composition, etc. Therefore, herders’ lives during their “last nomadic period” in Inner Mongolia provide an ideal system to evaluate and quantify the influences of political pressures on their vulnerability to climate hazards (Fig. 1). The objectives of this study are to:

1. compare the differences of climate condition between two sociopolitical periods in the entire Inner Mongolia region;

2. quantify, through rigorous statistical modeling and analyses, whether and how climate and sociopolitical factors influenced different livestock (large and small) population metrics.

## Methods

### Study Area and Background

**Figure 2:**
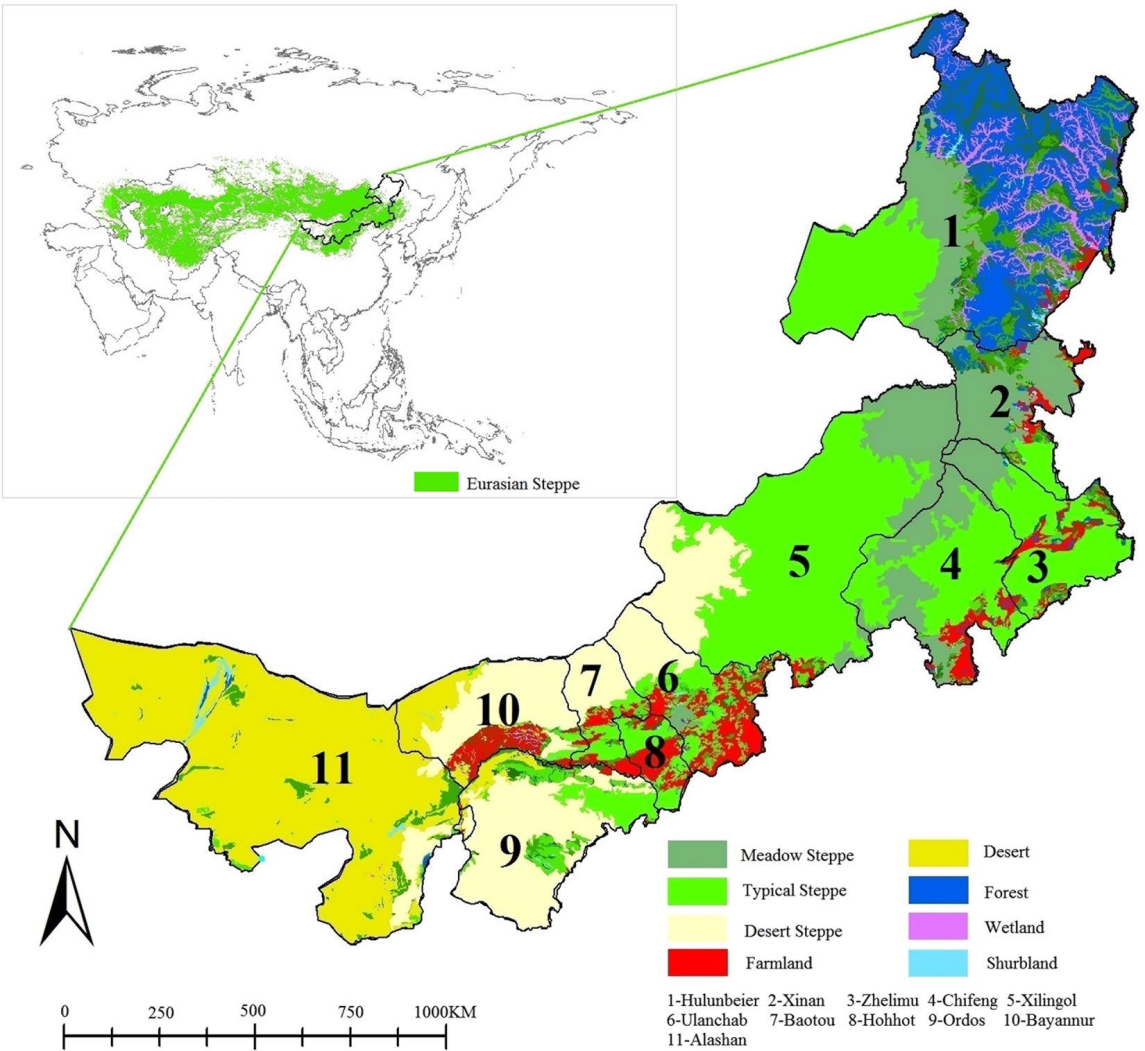
Geography, Vegetation Type and 11 Prefecture-Level Districts of Inner Mongolia. Note: Figure generated with ArcGIS 10.2 by authors (http://www.esri.com/software/arcgis/arcgis-for-desktop).

Inner Mongolia is located in the southeastern Eurasian steppes in northern China, covering a total area of 1.183 million km^2^ and spanning from 37°N to 53°N and from 97°E to 126°E. Inner Mongolia is divided into 11 administrative regions (Fig. 2). The geographical distributions of precipitation and temperature have shaped six eco-zones in Inner Mongolia, including forest, meadow steppe, typical steppe, desert steppe, desert, and agro-pastoral transitional zone. The annual precipitation, which gradually declines from east to west, is the major limiting factor of primary production of the ecosystem, except forest zone. Average annual precipitation is 390 mm in the meadow steppe, 330 mm in the typical steppe, and 220 mm in the desert steppe. Deserts located in the western part of Inner Mongolia have an average annual precipitation that is usually lower than 180 mm. The agro-pastoral transitional zone mainly spans the 400 mm isohyet line. During the last nomadic period, herders in Inner Mongolia raised five native animals, such as cattle, horses, camels, sheep, and goats. Traditionally, herders defined sheep and goat as small livestock, and the other three as large livestock. In the agro-pastoral transitional zone, herders also fed these five native animals on natural grassland, except for foraging crops, such as corns and alfalfa.

In Inner Mongolia, drought is the most adverse climate disaster in growing seasons (June-August). During periods of drought, plants offer less nutrients for livestock, causing animals to suffer from hunger, dehydration, and malnutrition. In winter, blizzards are the most inclement weather condition. The high mortality of animals in blizzards is not only due to direct frostbite and hypothermia, but also through long-term famine and calorie deficits when snow buries all edible parts of grasses. Additionally, low temperature threatens the life of neonate animal and can cause massive death during the breeding period in early season (March to April, depending on the eco-zones).

Herders and their livestock in Inner Mongolia experienced dramatic political pressures during the social movement period from 1964 to 1978, which could be further divided into four sub-periods: socialist education movement period (Stage I, 1964–1965, the prelude to the Cultural Revolution), the most chaotic anarchy period (stage II, 1966–1969), military controlling period (stage III, 1970–1976), and standstill period (stage IV, 1977–1978, the epilogue of the Great Cultural Revolution). These sub-periods mainly differed in sociopolitical pressures. The other years (1961–1963 and 1979–1986) are considered as the peaceful period in this study, where herders had much more freedom to raise and manage their livestock (Fig. 1).

### Data Collection, Preprocessing, and Exploratory Analysis

We retrieved the annual livestock demographic data for the five animals between 1961 and 1986 from the Inner Mongolian animal husbandry statistical yearbooks^27^. The spatial scale was at the sub-district level. The annual livestock population demographic metrics included adult mortality rate, neonatal mortality rate, and birthrate. During nomadic periods, these metrics were very sensitive to climate fluctuations^6^. Thus, this study used these three demographic metrics as indicators to evaluate nomadic animal husbandry vulnerability, and they were the dependent variables for the statistical modeling discussed later.

**Figure 3:**
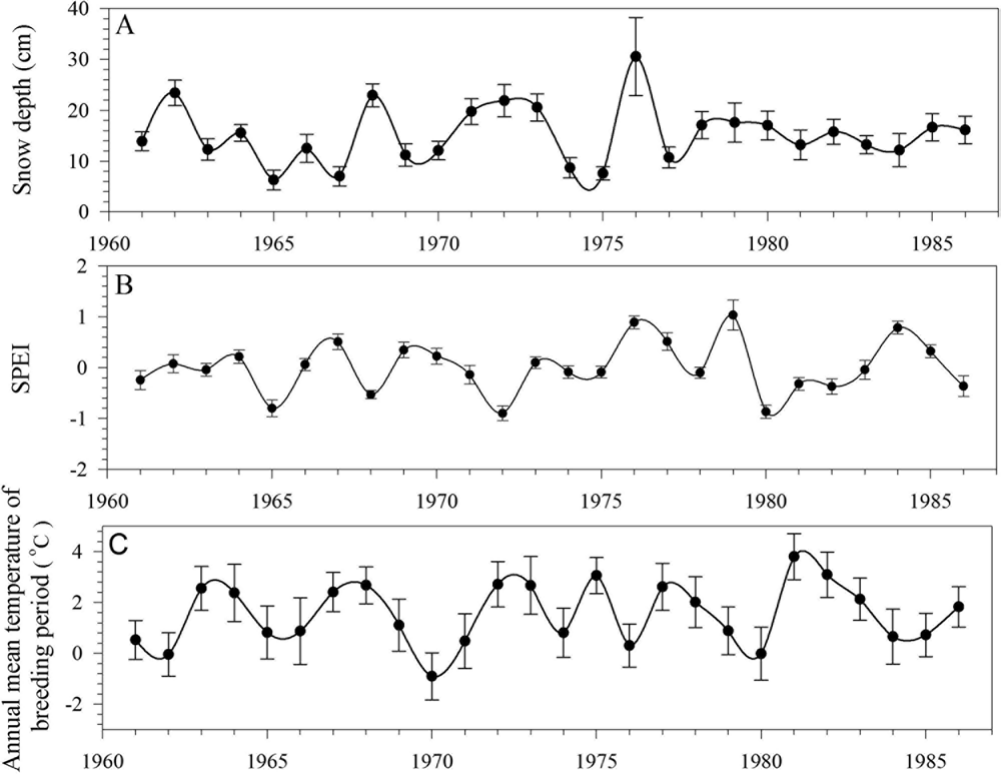
Overview of Climate Conditions between 1961 and 1986 in Inner Mongolia Note: SPEI stands for Standardized Precipitation Evapotransporation Index, a measurement of drought. Solid dot represents mean value, and error bars represent standard errors from the 11 districts/counties.

The climate data were acquired from the National Climate Data Service Center of China (http://data.cma.gov.cn/). We used un-melt snow depth (Fig. 3A), Standardized Precipitation Evapotransporation Index (SPEI) of growing season (Fig. 3B), and mean temperature of March and April (Fig. 3C) to represent and quantify three types of climate harshness: winter harshness, dry condition of growing season, and the cold stress in breeding season, respectively. SPEI were calculated by SPEI package (http://sac.csic.es/spei/) of *R* software^28^. These three metrics were the climate independent variables. In addition, We also retrieved annual mean temperature and growing season rainfall to demonstrate general climate conditions and patterns during the study period.

We further evaluated whether climate hazards occurred homogeneously during different periods (i.e., whether some sociopolitical periods had more frequent and intense climate hazards). This was important to differentiate and evaluate the effects of climate and sociopolitical factors. For instance, if climate hazards occurred more frequently in social movement periods, it would potentially alter the results. We investigated the pattern of climate hazards occurrence between peaceful and social movement periods. We defined a dry year as a year with its SPEI value below the 26-year (1961–1986) average minus its 1/2 standard deviation (mean-1/2 SD) for each sub-district. The cold breeding season year was defined as an early season temperature below the average of 26-year temperature minus its 1/2 standard deviation (mean - 1/2 SD). For snow factors, we considered a snowy year as a snow depth more than 26-yearaverage snow depth plus its 1/2 standard deviation (mean + 1/2 SD). We used the unbalanced one-way analysis of variance (ANOVA) to compare the intensity and frequency of adverse climate years in the two sociopolitical periods for three adverse climate hazards, and checked the requirements of ANOVA (i.e., normality, homoskedasticity) beforehand.

### Quantifying Effects of Climate and Sociopolitical Factors through Generalized Additive Model

We divided the entire study period (1961–1986) into two groups based on the sociopolitical pressure levels: social movement period group (1964–1978) and peaceful period group (1961–1963, and 1979–1986). We quantified and differentiated herder’s and their livestock’s vulnerability to adverse climate using generalized additive model (GAM) because the relationship between dependent variables and independent variables were presumably nonlinear. For instance, too much snowfall in the winter would cause death of livestock, but too little snowfall would also result in insufficient water supply during early spring which may result in livestock mortality. The response variables (dependent variables) were each of the three metrics: adult livestock mortality rate, neonatal livestock mortality rate, and livestock birthrate for both large and small animals. The independent variables (explanatory variables) included SPEI, snow depth, and winter precipitation, which represented climate metrics, as well as the two sociopolitical periods (peaceful and social movement period). We first constructed the full GAM, which included both climate metrics and sociopolitical metrics, and the potential interactions between each of the climate metrics and sociopolitical metric. We did not consider interactions between climate metrics themselves in this study. Consequently, the complete GAM was specified as:

1 $$\begin{array}{c}{y}_{j}={\beta }_{1j}{\rm{f}}({x}_{1j})+{\beta }_{2j}{\rm{f}}({x}_{2j})+{\beta }_{3j}{\rm{f}}({x}_{3j})+{\beta }_{4j}{\rm{f}}({x}_{4j})+{\beta }_{5j}{\rm{f}}({x}_{1j}{x}_{4j})\\ \quad \quad +{\beta }_{6j}{\rm{f}}({x}_{2j}{x}_{4j})+{\beta }_{7j}{\rm{f}}({x}_{3j}{x}_{4j})+{\beta }_{0j}\end{array}$$

Where *y*_*j*_ represented one of the three dependent variables (neonatal mortality, adult mortality, birthrate, *j* = 1, 2, 3); *x*_*1j*_, *x*_*2j*_, and *x*_*3j*_ represented three climate metrics, and *x*_*4j*_ represented the sociopolitical metric. Since this is GAM, *f* (*x*_*ij*_) indicated a nonlinear relationship for independent variables (i.e., the response was not linear). *β*_*1j*_ through *β*_*7j*_ were the coefficients associated with the GAM terms, and *β*_*oj*_ was the theoretically normally distributed random error. Large and small animals were coded as a binary term (not shown in Eq. 1, specified in *R* script). We also considered potential temporal auto-correlation in the dependent variable in this full model (not shown in Equation 1, specified as the first-order autoregressive term in *R* script).

Then, we tested two more simplified GAMs: the first one did not include the interaction terms in the full model (corresponding to *β*_*5j*_, *β*_*6j*_, *β*_*7j*_ terms, i.e., no interaction between sociopolitical and climate metrics), hypothesizing that sociopolitical and climate metrics were uncorrelated and decoupled. The other further simplified model did not include sociopolitical factors at all, serving as the null model that hypothesized herders’ animal husbandry was not affected by external sociopolitical pressures.

Two frequently used coefficients were adopted for model selection: the Akaike information criterion (AIC) and coefficient of determination (DEC)^29^. The generalized cross validation score (GCV) was also provided^30^. The optimal model among the three (one complete and two simplified GAMs) was chosen based on the smallest AIC and GCV value and the largest DEC as well. If the numerical value of AIC values did not differ substantially (i.e., the difference was smaller than 10 among AIC values), we generally acknowledged there was no difference among the corresponding models^31^. When the optimal model was identified, we reported the coefficient value (*β*) associated with each term in the optimal model, including the potential interaction terms. We also provided the diagnostics of the optimal GAM, including Q-Q plots, histogram of residuals to demonstrate the model goodness-of-fit.

Although the animal husbandry data were published by government agencies, the quality of these data was not assessed. In order to investigate the robustness of our model against potential observation and system errors, we performed a sensitivity analysis by adding white noise to the data, and re-evaluated the parameter estimations from the new dataset.

### Quantifying and Comparing the Effects of Sociopolitical Pressure during Different Periods

As shown later in the results section, sociopolitical pressure was indeed a critical factor in livestock vulnerability to climate hazards. Besides, climate harshness (measured by three climate hazards) was similar during different periods (also demonstrated later in the results section). We investigated whether herder’s livestock mortality and birthrate (for both large and small animals independently) differed significantly between the two periods in relation to two sets of climate hazards: early spring cold stress and growing season drought (measured by SPEI), using an unequal sized *t*-test. Snow depth was not included because this factor alone never had significant impact on livestock metrics according to the GAM results. The comparison could reveal and quantify the severity that political pressures had placed on herders’ lives in animal husbandry.

In addition to the comparison between the peaceful and social movement periods, we also further quantified livestock demographic metrics in the four different stages of the social movement periods, and investigated whether herders were able to adapt to the political pressures.

All Statistical analyses in this study were carried out in *R* software (Version 3.2.4) with *mgcv*, *itsadug* and *nlme* packages^32^. All scripts and original data are freely available upon request.

## Result

### Climate Condition Similar during Study Period

The three climate metrics (snow depth, SPEI, and early spring temperature) did not show any substantial change between the social movement period and the peaceful period (Fig. 3). There were no significant differences in annual mean temperature (*p* = 0.72) and annual growing season rainfall (*p* = 0.55) between the two periods. The dry years in the peaceful period were more frequent than in the social movements period, but the difference was not significant on a sub-district scale either (*p* = 0.06, Fig. 3B). The frequencies of heavy snowfall and cold breeding seasons were similar in the two periods too (Table 1, Fig. 3B).

**Table 1:** Comparison of Frequency and Intensity of Three Climate Hazards in Social Movement period and peaceful period.

Climate Metrics		Social Movements period	Peaceful Period	F_df1, df2_	*p-*value
Average	GP (mm)	209.96(23.89)	205.70(24.06)	0.36_1, 229_	0.5501
	TB(°C)	1.29(0.99)	1.36(1.00)	0.13_1, 229_	0.7198
Frequency (%)	DY	24.6(3.4)	35.4(3.4)	4.79_1, 9_	0.0565
	HSY	27.3(7.1)	28.1(7.1)	0.06_1, 9_	0.8124
	CBSY	26.6(1.17)	28.1(1.7)	0.8_1, 9_	0.3955
Intensity	SPEI	−0.69(0.05)	−0.73(0.05)	0.29_1, 65_	0.5908
	SD(cm)	26.57(0.96)	24.65(1.10)	1.85_1, 62_	0.1792
	TB(°C)	−2.48(0.66)	−2.26(0.68)	0.43_1, 65_	0.5137

Note: GP, TB, DY, HSY, CBSY, SPEI, SD, and CBP stand for growing season precipitation, annual mean temperature, dry years, heavy snowfall years, cold breeding season year, standardized precipitation evapotranspiration index, snow depth, and cold breeding season events respectively. There were no significant differences in climate conditions between peaceful and social movement periods.

The average intensity of the three climate factors did not show any significant difference either. SPEI of drought events was −0.69 ± 0.05 in the social movement period, and −0.73 ± 0.05 in the peaceful period (*p* = 0.59). The annual snow depth in snowy years was 26.57 ± 0.96 cm in the social movement period, and 24.65 ± 1.10 cm in the peaceful period. The difference was insignificant also (*p* = 0.17). The temperature of cold breeding season years were −2.48 ± 0.69 °C, and −2.27 ± 0.68 °C in the social movement period and the peaceful period respectively. There was no significant difference between them (*p* = 0.51, Table 1, Fig. 3B). Thus, general climate conditions as well as hazard climate events occurred homogeneously during the study period in Inner Mongolia, and any potential changes in livestock demographic metrics should be attributed more substantially to sociopolitical factors and their interactions with climate.

### Livestock Population Metrics Fluctuated in Different Periods

**Figure 4:**
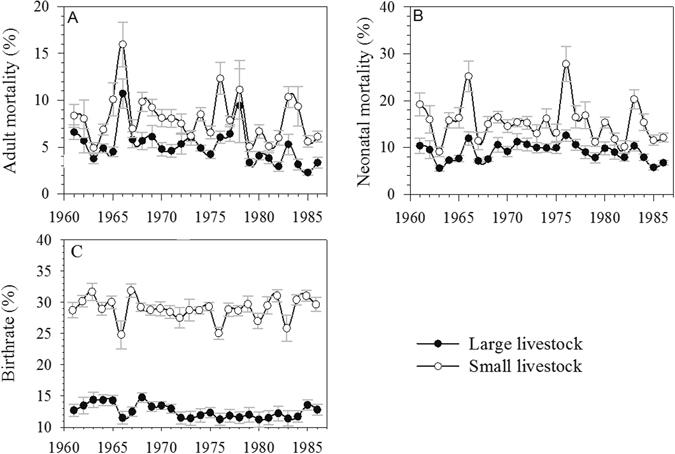
Overview of Livestock Population Dynamics between 1961 and 1986 in Inner Mongolia Note: Metrics were demonstrated on sub-district scale of Inner Mongolia: adult mortality (**A**), neonatal mortality (**B**), and birthrate (**C**). Black solid dot stands for large livestock, such as cattle, horse, and camel, while hollow dot stands for small livestock, such as sheep and goat. Solid dot represents mean value, and error bars represent standard errors from the 11 districts/counties.

Herders’ animal husbandry experienced long-term depression during the entire social movement period (1964–1978). Average annual adult mortality rate of large livestock in the social movement period was 5.87%, which was 1.5 times higher than in the peaceful period. Average adult mortality of small livestock was 8.94%, which was 1.29 times higher than in the peaceful period (Fig. 4A). As for the neonatal mortality, it was 9.74% and 16.28% for large and small livestock respectively in the social movement period, which were also significantly larger than the two metrics in the peaceful period (7.97% for large and 13.74% for small animal, Fig. 4B). Also, the birthrates of large and small livestock during the social movement period were 12.46% and 28.58%. These metrics were also marginally higher: 12.70% for large and 29.55% for small animals in the peaceful period (Fig. 4C). Thus, adult and neonatal mortality rates (for both small and large animals) were influenced more substantially during the social movement period, but birthrates seemed to be less disturbed through different periods.

### GAM Identified Critical Role of Political Pressures

The full model for adult mortality was the optimal model based on AIC, GCV, and DET values (Table 2), and the models without interaction between climate and sociopolitical factors, as well as the models without sociopolitical factors were both sub-optimal. The results in the full, optimal model showed that SPEI (Standardized Precipitation Evapotransporation Index) temperature in early spring (March to April), and sociopolitical factors significantly influenced adult mortality, and large and small animals also differed significantly for adult mortality (Table 3). Additionally, interactions between SPEI and sociopolitical factors, as well as the interactions between snow depth and sociopolitical factors were significant too. These results suggested that SPEI and snow depth could intermingle with sociopolitical factors (political pressures), while early spring temperature did not interact with political pressures. Interestingly, this full model did not detect the significant effects of snow depth alone on adult mortality in Inner Mongolia. The diagnostic plots of the GAM showed that the model fit reasonably well according to the Q-Q plot and residual plot. These results are provided as Supplementary information (SI Fig. 1) with this manuscript.

**Table 2:** Goodness-of-Fit of Models for Three Population Demographic Metrics.

Models for adult mortality	GCV	DET	AIC
Full model (include climate factors, social factor and interactions)	11.67	86.9%	2841.68
Model 1 (include climate factors, and social factor)	11.86	49.8%	2921.56
Model 2 (only include climate factors)	12.69	45.5%	2959.18
**Models for neonatal mortality**	**GCV**	**DET**	**AIC**
Full model (include climate factors, social factor and interactions)	10.33	60.3%	2841.68
Model 1(include climate factors, and social factor)	32.23	34.3%	3471.97
Model 2 (only include climate factors)	33.33	31%	3490.67
**Models for Birth rate**	**GCV**	**DET**	**AIC**
Full model (include climate factors, social factor and interactions)	11.67	86.9%	2913.02
Model 1 (include climate factors, and social factor)	11.99	86.1%	2928.43
Model 2 (only include climate factors)	12.14	85.7%	2935.67

Note: AIC indicates Akaike information criterion (smaller for better model), GCV stands for generalized cross validation score (smaller for better model), and DEC denotes coefficient of determination (larger for better model).

**Table 3:** GAM Results for Climate and Sociopolitical Factors in Full Models.

Factors in GAM	*E*df	Ref.df	*F*	*p*-value
**Adult Mortality**				
Year	7.89	8.67	2.79	**0.0064**
SPEI	5.81	6.95	2.67	**0.0112**
Snow	1	1	0.50	0.4802
TMA	1	1	17.44	**<0.0001**
Social	3.89	3.98	10.04	**<0.0001**
SPEI*Social	9.86	11.53	2.34	**0.0072**
Snow*Social	12.72	13.82	19.44	**<0.0001**
TMA*Social	8.96	11.29	1.76	0.0547
**Neonatal Mortality**				
Year	7.99	8.62	2.67	0.0729
SPEI	7.84	8.62	2.69	**0.0048**
Snow	1	1	1.13	0.2868
TMA	2.06	2.63	3.76	**0.0133**
Social	3.77	3.96	4.42	**0.0015**
SPEI*Social	3.61	4.96	0.66	0.6410
Snow*Social	12.68	13.69	2.63	**0.0012**
TMA*Social	1	1	1.76	0.1853
**Birthrate**				
Year	1.65	2.08	0.53	0.5717
SPEI	3.17	3.98	2.69	**0.0274**
Snow	1	1	0.34	0.5577
TMA	1	1	54.46	**<0.0001**
Social	3.77	3.96	3.20	**0.0232**
SPEI*Social	8.86	10.76	1.88	0.0742
Snow*Social	1.39	1.71	0.20	0.6954
TMA*Social	1	1	0.989	0.3206

Note: full models were optimal models for each demographic metric. SPEI, Snow, and TMA corresponded to winter harshness, drought, and growing season harshness. Bald values indicate significance (<0.05).

In terms of neonatal mortality, the full model was also superior to the two other simplified models. SPEI, temperature of early spring, sociopolitical factors and the interaction between snow depth and sociopolitical factors had significant influences, and there was significant difference between large and small animals (Table 2). Again, snow depth alone did not contribute much to neonatal mortality, but the interaction between snow depth and sociopolitical factors was much more important, according to the GAM results. Inspection of the diagnostic plots showed that this GAM also worked well (SI Fig. 2).

Lastly, SPEI, temperature of early spring, locations, and sociopolitical factors significantly influenced birthrates (Table 2). However, no interactions between these variables had any significant impact on birthrate. In fact, the three GAM worked almost equally well for birthrate (Table 2). This indicated a decoupling between climate and sociopolitical factors for birthrates for both large and small animals. The diagnostic plots confirmed the validity of this GAM (SI Fig. 3).

These results demonstrated that sociopolitical factors had significant impacts on all three demographic metrics for both large and small animals; however, the major contributing factors for different metrics were very different. According to the sensitivity analysis, the results were stable under input data disturbance (i.e., observation error). We concluded from the GAM results that political pressures had played an important role in animal husbandry and indeed intensified herders’ and their livestock’s vulnerability to adverse climate conditions.

### Political Pressures Increased Livestock’s Vulnerability to Climate Hazards

**Figure 5:**
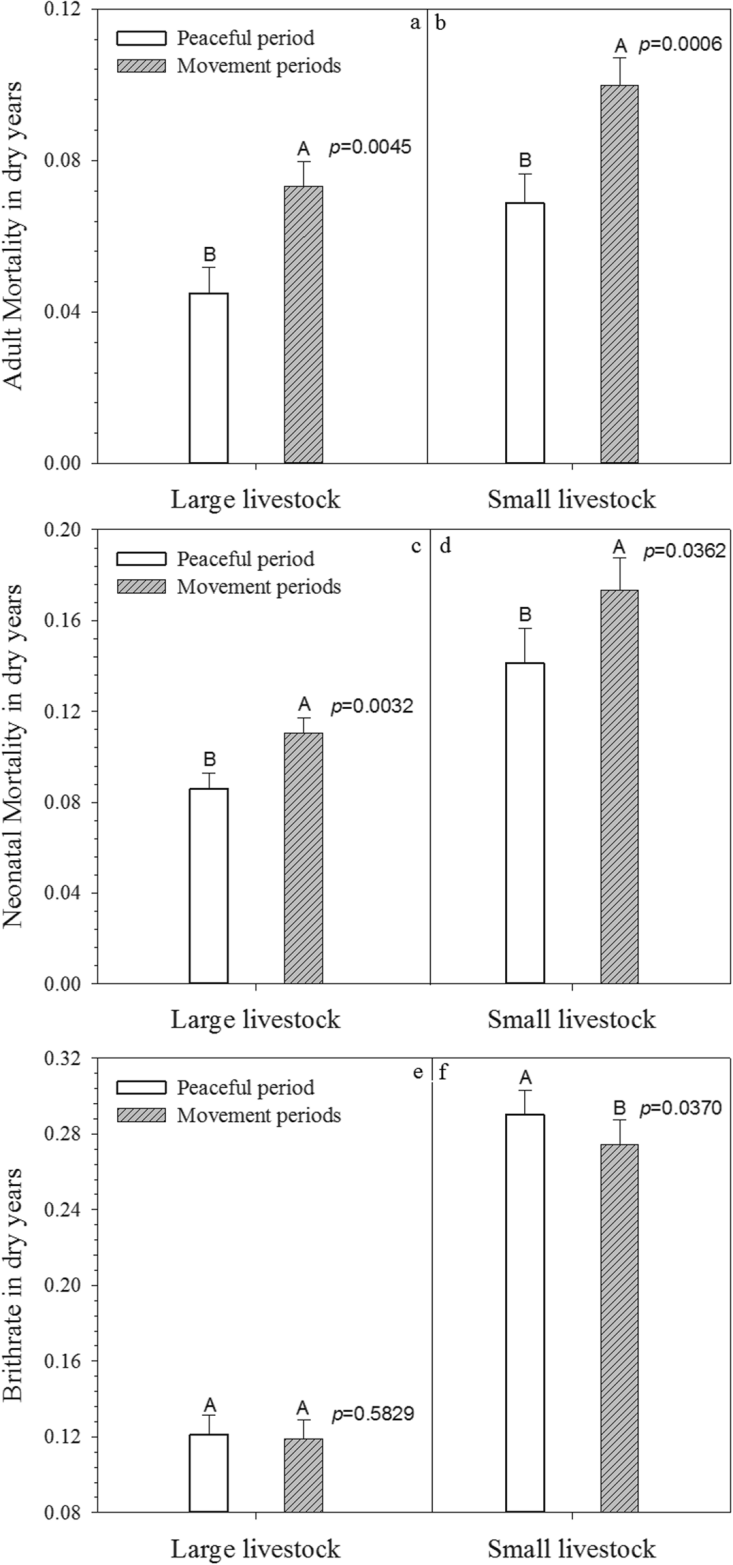
Comparison of Adult, Neonatal Morality and Birthrate in Drought Years during Peaceful and Social Movement Periods. Note: error bars represented standard errors. All population demographic metrics showed significantly differences between peaceful and social movement periods, except birthrate for large livestock.

**Figure 6:**
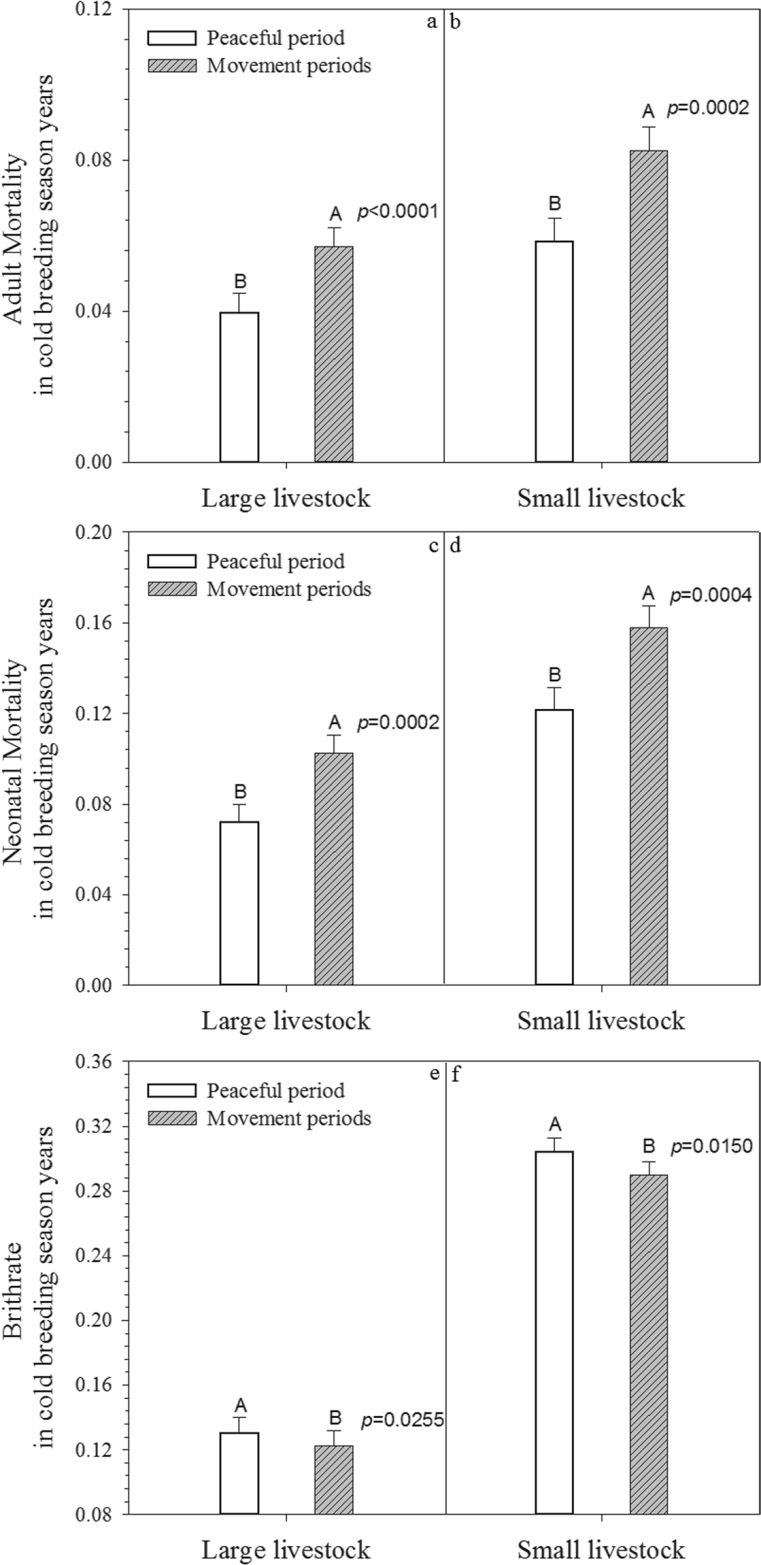
Comparison of Adult, Neonatal Morality and Birthrate in Cold Years during Peaceful and Social Movement Periods. Note: error bars represented standard errors. All population demographic metrics showed significantly differences between peaceful and social movement periods.

We concluded from the GAM results that social movement and political pressures significantly impacted both large and small livestock’s population demography, and we further demonstrated livestock’s vulnerability to climate hazards. We focused on drought (Fig. 5) and early breeding season coldness (Fig. 6) and omitted snow because snow alone did not significantly impact livestock populations, according to the GAM results.

Both large and small livestock’s adult mortality rate was significantly higher in social movement drought years than in peaceful period drought years (Fig. 5A,B), and the same was true for both large and small livestock’s neonatal mortality (Fig. 5C,D). Although there was no statistical difference in large livestock’s birthrate during drought years between the two periods (Fig. 5E), small livestock’s birthrate was significantly lower in social movement drought years (Fig. 5F). This could be due to the fact that some large animals (e.g., horses) were considered for military use and horse reproduction was much less influenced.

Excessive coldness in early spring was another major climate hazard for both large and small livestock: all three demographic metrics (adult mortality, neonatal mortality, and birthrate) showed significant differences in excessive cold spring years between the two periods (Fig. 6A–F), and social movement years coupled with excessive cold always yielded significantly unfavorable livestock populations.

Besides the substantial difference between the social movement period and the peaceful period, we also demonstrated how livestock demographic metrics changed within four sub-stages of the social movement period. The complete results are shown in SI Fig. 4 to SI Fig. 6. Adult mortality and neonatal mortality showed a substantial increase during the first stage of the social movement period, and slowly but steadily rose back towards the normal level as in the peaceful period. Birthrate followed a similar pattern for small animals: it decreased substantially during the first stage and bounced back towards the end of the social movement period. However, for large animals, birthrate did not show substantial change, and actually decreased a bit during the third and fourth stages, where all other demographic metrics usually recovered. The changes of all demographic metrics, except birthrate of large livestock, indicated that herders could adapt to the social movements and cope with high political pressures, except in managing large livestock.

## Discussion

In this study, we combined a unique time series dataset with rigorous statistical analyses to quantify the effects of climate and sociopolitical factors (and their potential interactions) on herders’ livestock population demography in Inner Mongolia, China. This study provided qualitative interpretations and new insights about the political pressures and nomadic herders’ vulnerability to natural hazards^8, 9, 17, 18^. We demonstrated that the climate condition and hazard occurrence did not differ substantially between the peaceful and the social movement periods; however, livestock (both large and small, adult and neonatal) mortality and birthrate changed substantially between these two periods, and livestock were much more vulnerable to climate hazards during the social movement period. The model results confirmed that political pressures, rather than natural harzards, significantly impacted nomadic livestock.

Excessive snowfall, called “*dzud*” by local herders, was considered the most intimidating natural disaster in the Mongolia plateau^6, 19^. Interestingly, snow depth alone was never a significant factor for livestock population fluctuation according to the GAM. The other two climate hazards, drought and a low temperature during the breeding season, had significant impacts on livestock, which is consistent with our current knowledge. We suggested that the paradox of snow depth was due to the spatial data resolution and spatial heterogeneity in this study. Inner Mongolia spanned six different eco-zones^33^; in forests, the plants are very tall to prevent them from being completely covered even after heavy snow fall, thus providing edible food for the nomadic livestock. For more dry desert and desert-steppe eco-zones, higher volume of winter snow would benefit livestock water supply in winter, as well as allow for better plant development in the following spring when snow melted. Heavy snow was only disastrous in typical steppes and meadow steppe (in Xilingol and northern Chifeng) where snow completely covered grasslands and killed a large amount of livestock. Thus, snow depth was not a “global” homogeneous risk factor across all regions in Inner Mongolia, but a more local/regional contributor, especially in steppe areas. This was consistent with previous studies in Mongolia country, where snowfall was only influential on a local scale, not regional scale^10^. In contrast, drought was a global restriction factor for the ecosystem primary production in Inner Mongolia^34, 35^, and a breeding season’s low temperature would also influence animal metabolism and physiology across all regions through Inner Mongolia^36^, so it was not surprising that these two factors impacted livestock population more homogenously in Inner Mongolia.

Similarly, political pressures (such as during the social movement period in Inner Mongolia) limited herders’ freedom in animal husbandry. In the peaceful period, herders relied on many useful traditional ecological experiences to resist the climate hazards, such as keeping the mobility of livestock^19^, raising high ratio of adult animals^8^, adjusting pregnancy rate, and culling certain weak neonatal individuals during hazard years^9^. These traditional strategies and practices provided the optimal way to survive in the harsh and unpredictable environment. In the peaceful period, local officials frequently sought advice from community elders, and made production decisions based on the actual vegetation and animal conditions^8^.

However, herders were deprived of these entitlements by politicians during the social movement period (1964–1978). Local elites lost freedom, and were marginalized due to the socialism education movement (1964–1965). Outsider revolutionaries had made many arrogant policies in livestock husbandry and management^25^. These outside leaders used some modern methods to replace the traditional practice of local herding. These livestock practices included improving pregnancy rate, improving the proportion of young individual, etc^37^. Although they were economically efficient for modern livestock husbandry, they were impractical in the nomadic periods^9, 17^.

The GAM results in this study demonstrated that both large and small livestock’s adult and neonatal mortality was significantly impacted by the interaction between climate and sociopolitical factors. We suggested that such interactions intensified livestock vulnerability to climate hazards. Although snow depth alone did not significantly impact livestock, its interaction with sociopolitical factors had substantial effect. In the nomadic era, mobility was the most important strategy of herders to cope with snow disaster and drought. However, herders lost their entitlement to freely decide when, where and how to move during the social movement period. The entitlement differences of maintaining mobility between the two periods caused the interactions between climatic factors and sociopolitical factors. However, birthrate was not influenced by the interaction, because herders always choose sunny and warm concave ground as breeding place and seldom move away during the whole breeding period.

We propose this methodological study with a real-world application. There are, however, potential alternative approaches. One of them evaluates the influences of social status as two—steps analysis. The first step is to set up the model between livestock metrics and climatic parameters in two periods separately; then, the next step is to compare the model parameters. We argue that our method is more favorable and practical than the alternative. First, many confounders, such as locust outbreaks and dust storms, could synergistically influence the model output. We cannot distinguish the contribution of political pressure from other confounders in the alternative method. Second, the two models are fitted from different datasets while our method utilizes the same dataset, which reduces systematic errors. Additionally, our proposed GAM approach handles nonlinear relationships more efficiently.

We admit that the dichotomy of the entire period into “peaceful” vs “social movement” is seemingly coarse. However, the major objective and focus of this study is not to investigate the detailed sociopolitical changes, which has been intensively explored and studied by various scholars in many qualitative studies. As an interdisciplinary study, we are more interested in extending our qualitative knowledge to more of a broad scale with robust quantitative methods. It is a common and well-acknowledged practice to simplify variables in quantitative studies. Many quantitative history studies are based on the dichotomy of variables, for example, the study on Japanese invasion^38^, Jewish persecutions^39^, European wars in medieval era^40^, revolts in Nile watershed^41^, and the great famine of China^42^. Therefore, simplifying political information with a dichotomical variable is a universal, practical, and informative method.

We are concerned about the data quality and its potential influence on this study. Animal husbandry was the cornerstone of Inner Mongolian economics prior to 1987. During the period of the government-planned economy, the state and local government took comprehensive records on all aspects of animal husbandry^37^. The nomadic people and the government had to cross check and confirm their data, because the data were the basis of the income for the nomadic people. The management of animal husbandry during the Cultural Revolution period is paradoxically even stricter – those who were responsible for intensive animal loss would be charged with felony (e.g., being the public enemy). Furthermore, some animals, especially large animals such as horses, were in military use to prepare for the potential invasion from USSR, thus the census of large animals was even more accurate. Inner Mongolian experts and scholars had reviewed these data under a peaceful political environment^43^. Therefore, even though there might be some census errors, these data are reasonably credible. Ultimately, sensitivity analysis showed that our conclusion was not substantially disturbed by data quality.

## Electronic supplementary material

Supplementary Information
