# Supplementary material for: Political Pressures Increased Vulnerability to Climate Hazards for Nomadic Livestock in Inner Mongolia, China
Source: Sci Rep. 2017 Aug 15;7:8256. doi: 10.1038/s41598-017-08686-4 (PMC5558013; doi:10.1038/s41598-017-08686-4)
Supplement: Supplementary file 1 — Supplementary Information [file 41598_2017_8686_MOESM1_ESM.pdf]

Title Page of Supplementary Information

SREP-16-33880A

Political Pressures Increased Vulnerability to Climate Hazards for Nomadic Livestock in Inner Mongolia, China

Ang Li, Shi Chen, Xueyao Zhang, Jianhui Huang

Figure SI legend

Panel 1: GAM goodness-of-fit diagnostic for adult mortality
Panel 2: GAM goodness-of-fit diagnostic for neonatal mortality
Panel 3: GAM goodness-of-fit diagnostic for birthrate
Panel 4: Adult mortality comparison in different periods
Panel 5: Neonatal mortality comparison in different periods
Panel 6: Birthrate mortality comparison in different periods

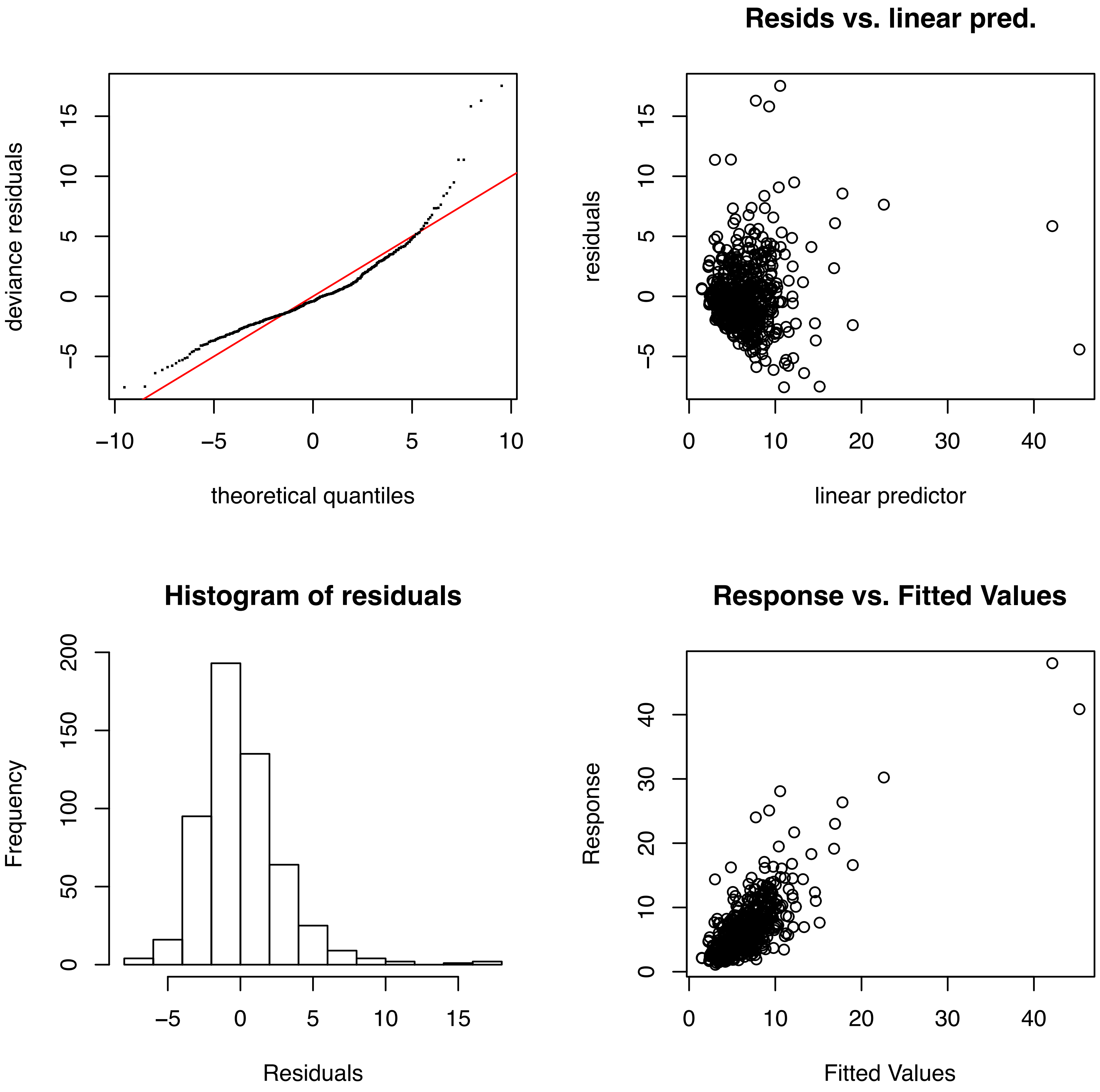

deviance residuals
theoretical quantiles
Resids vs. linear pred.
residuals
linear predictor
Histogram of residuals
Frequency
Residuals
Response vs. Fitted Values
Response
Fitted Values

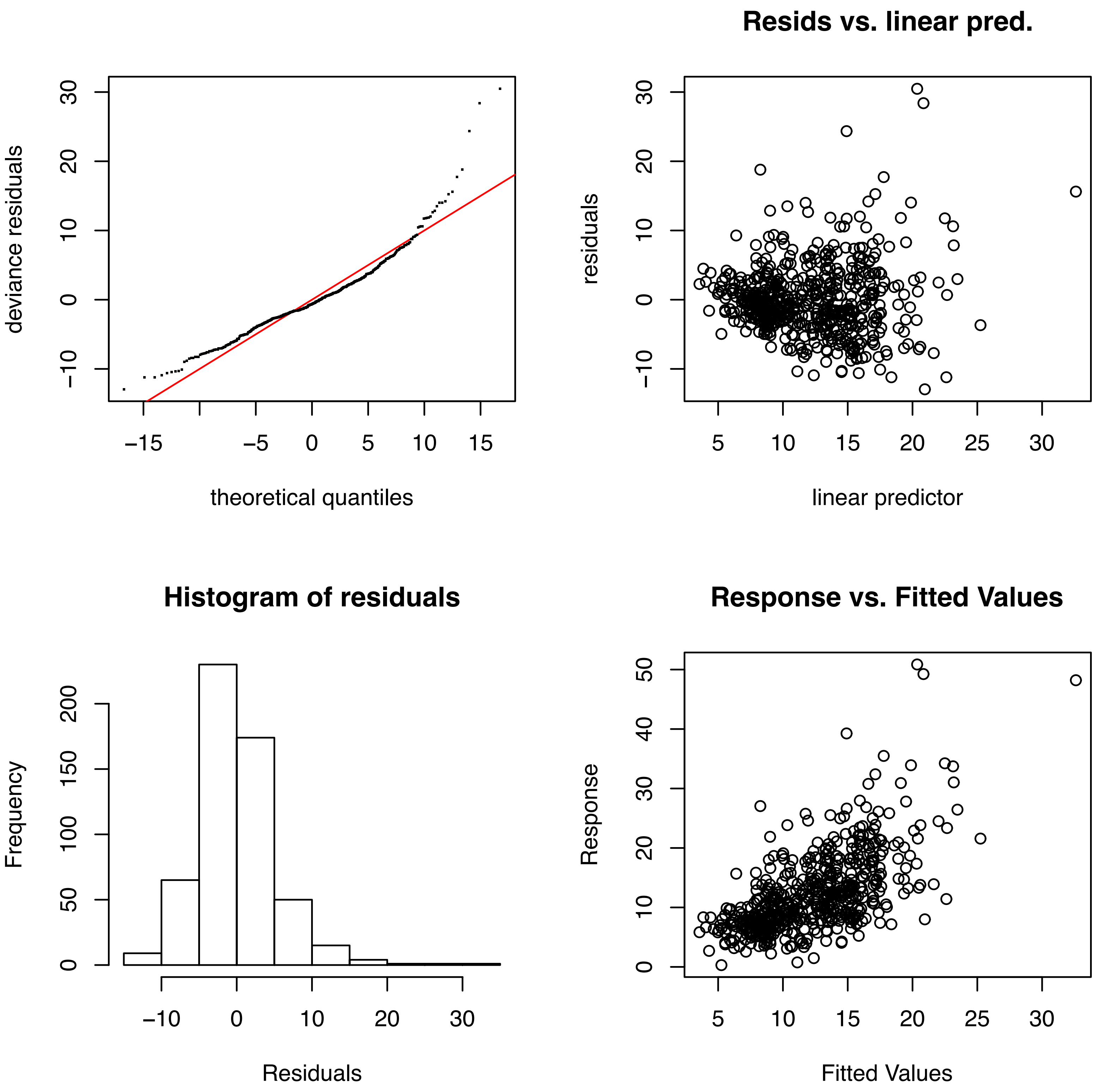

deviance residuals
theoretical quantiles
Resids vs. linear pred.
residuals
linear predictor
Histogram of residuals
Frequency
Residuals
Response vs. Fitted Values
Response
Fitted Values

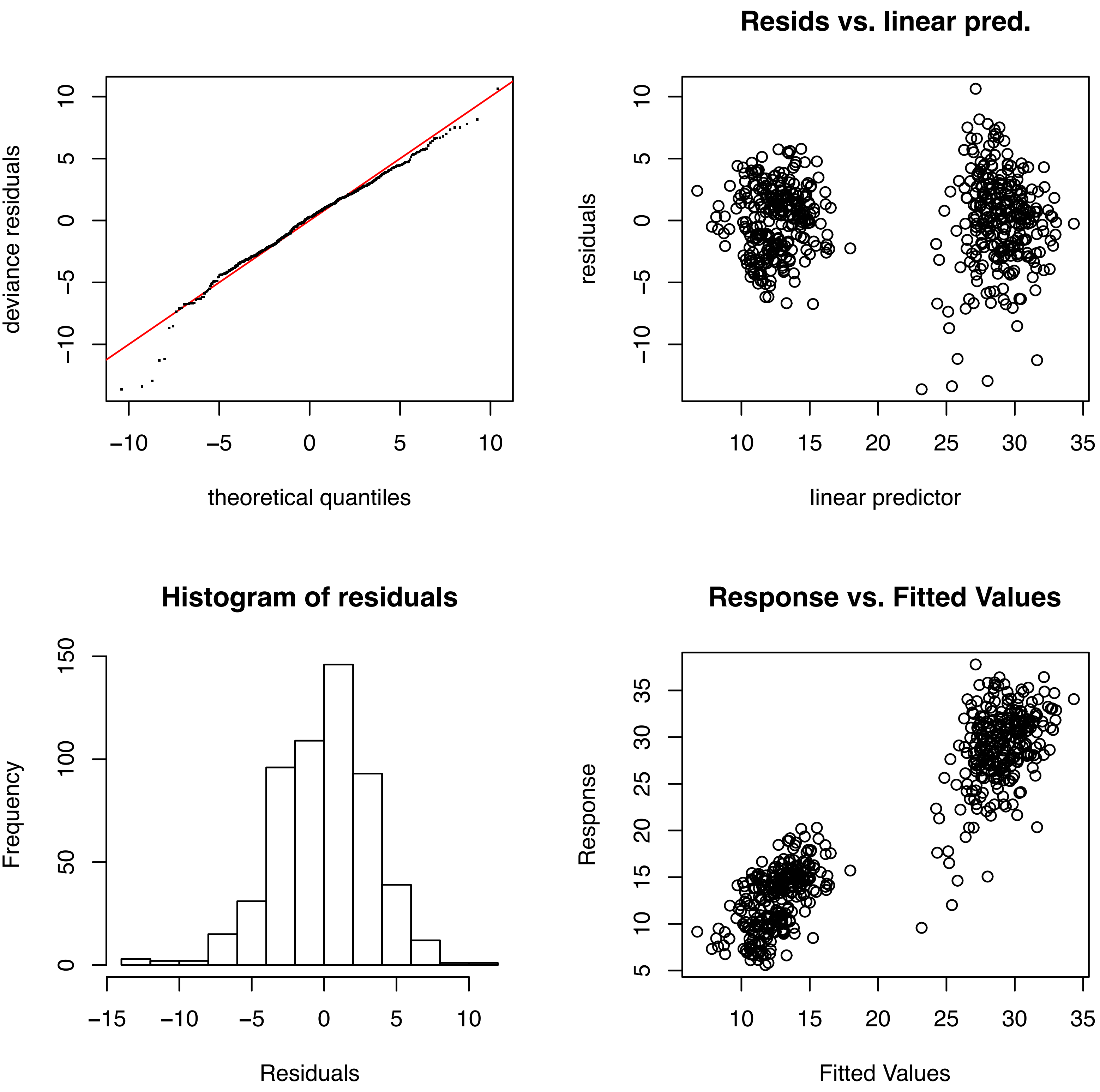

Resids vs. linear pred.
deviance residuals
theoretical quantiles
residuals
linear predictor
Histogram of residuals
Frequency
Residuals
Response vs. Fitted Values
Response
Fitted Values

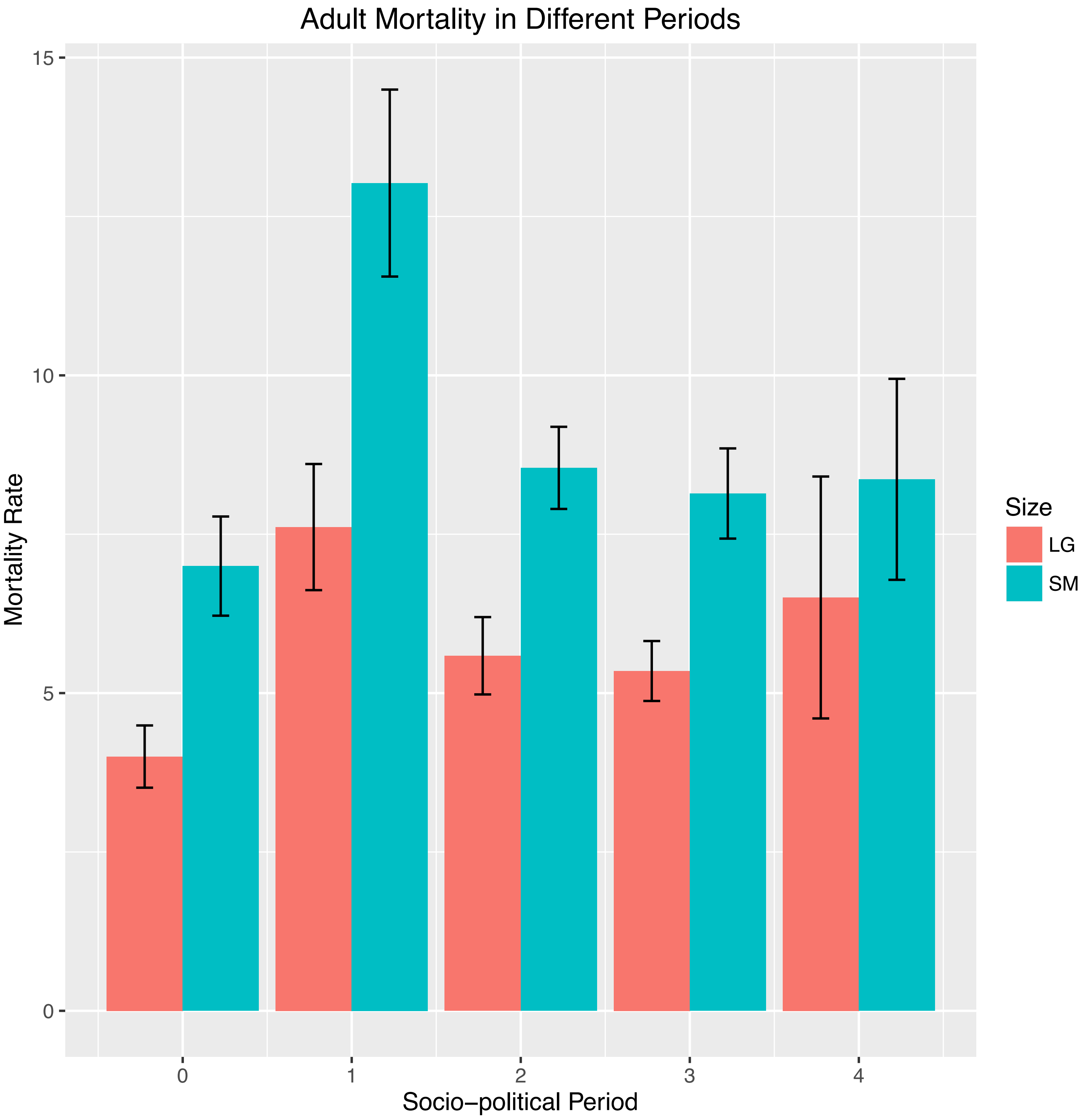
Adult Mortality in Different Periods
Mortality Rate
15
10
5
0
0
1
2
3
4
Socio−political Period
Size
LG
SM

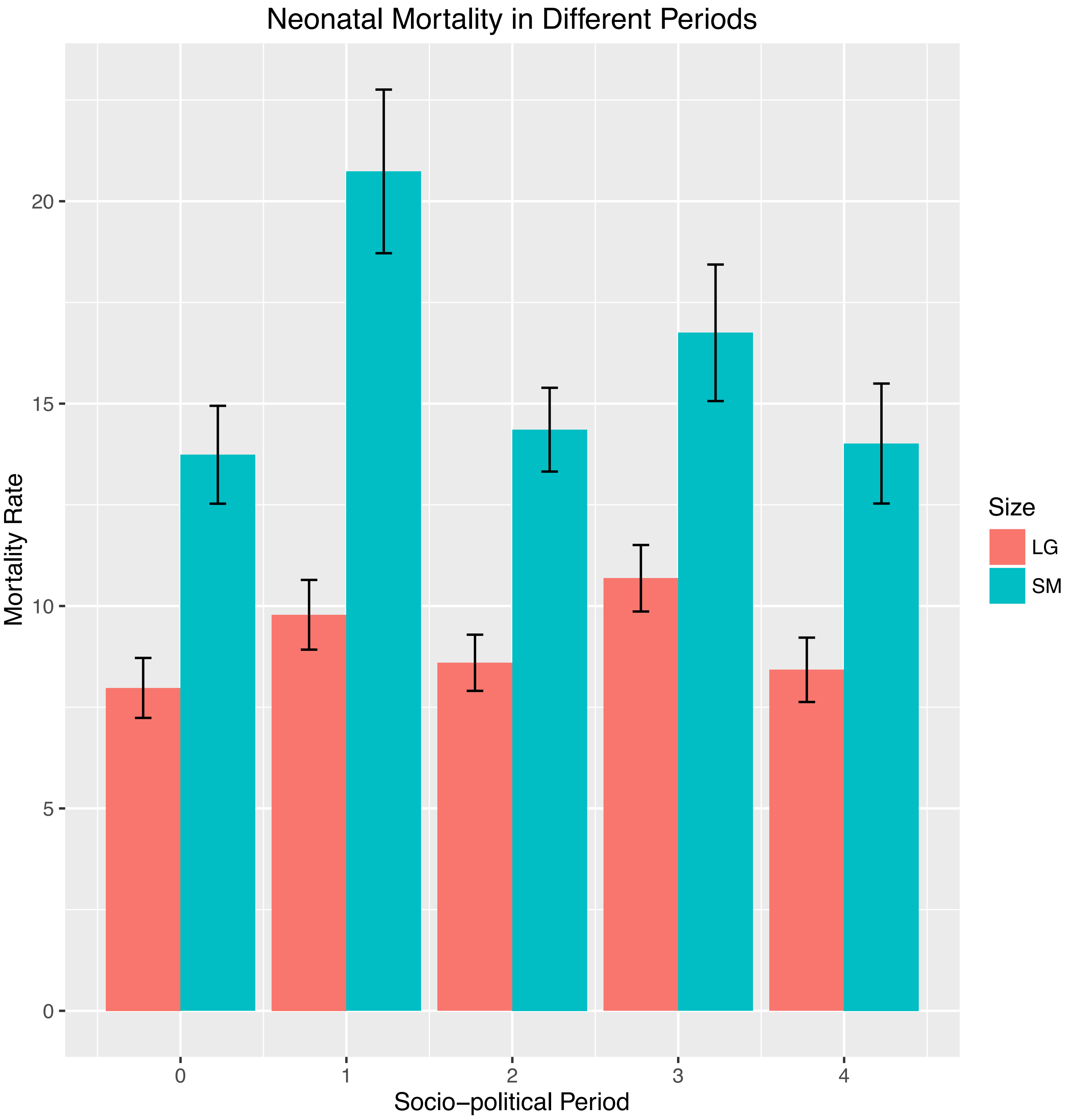
Neonatal Mortality in Different Periods
Mortality Rate
0
5
10
15
20
0
1
2
3
4
Socio−political Period
Size
LG
SM

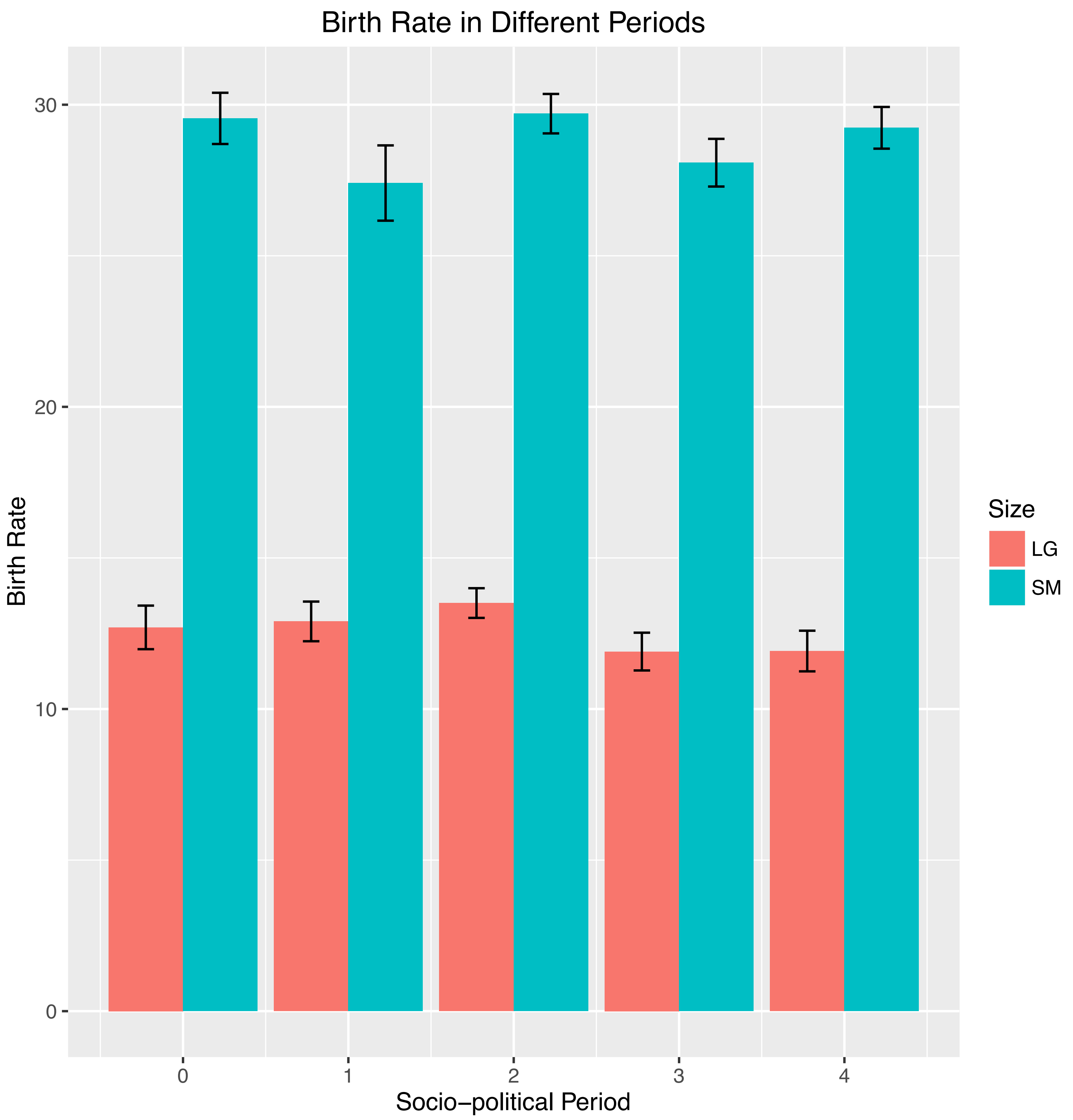

Birth Rate in Different Periods
Birth Rate
30
20
10
0
0
1
2
3
4
Socio−political Period
Size
LG
SM
